# Effect of physician attire on patient perceptions of empathy in Japan: a quasi-randomized controlled trial in primary care

**DOI:** 10.1186/s12875-021-01416-w

**Published:** 2021-03-31

**Authors:** Takaharu Matsuhisa, Noriyuki Takahashi, Kunihiko Takahashi, Yuki Yoshikawa, Muneyoshi Aomatsu, Juichi Sato, Stewart W. Mercer, Nobutaro Ban

**Affiliations:** 1 Department of General Medicine/Family & Community Medicine, Nagoya University Hospital, Tsurumai-cho 65, Showa-ku, Nagoya, 466-8560 Japan; 2 Department of Education for Community-Oriented Medicine, Nagoya University Graduate School of Medicine, 65 Tsurumai-cho, Showa-ku, Nagoya, 466-8560 Japan; 3 M&D Data Science Center, Tokyo Medical and Dental University, 1-5-45 Yushima, Bunkyo-ku, Tokyo, 113-8549 Japan; 4 Department of Medical Education, Saku Central Hospital, 197 Usuda, Saku, 385-0051 Japan; 5 Department of General Medicine/Family & Community Medicine, Nagoya University Graduate School of Medicine, 65 Tsurumai-cho, Showa-ku, Nagoya, 466-8560 Japan; 6 Centre for Population Health Sciences, Usher Institute, University of Edinburgh, Old Medical School, Teviot Place, Edinburgh, EH8 9AG Scotland; 7 Medical Education Center, Aichi Medical University School of Medicine, 1-1 Yazakokarimata, Nagakute, 480-1195 Japan

**Keywords:** Empathy, Physician attire, CARE Measure, Primary health care, Patient–physician relationship, Quality of care

## Abstract

**Background:**

There is limited quantitative research on the effect of physician attire on patient–physician relationships. This study aimed to measure the influence of Japanese family physicians’ attire on the “human” aspects of medical care in terms of patient-perceived relational empathy.

**Methods:**

This was a multicenter, prospective, controlled trial conducted in primary clinics in Japan. We explored the effects of family physician attire (white coat vs. casual attire) on patient-perceived empathy. Family physicians were allocated to alternate weeks of wearing a white coat or casual attire during consultations. Patients’ perceptions of physician empathy were evaluated using the self-rated Japanese Consultation and Relational Empathy (CARE) Measure. We used a linear mixed model to analyze the CARE Measure scores, adjusting for cluster effects of patients nested within doctor, age, and sex of patients, and doctors’ sex and years of clinical experience. We used the same method with Bonferroni adjustment to analyze patient sex differences in perceived empathy.

**Results:**

A total of 632 patients of seven family physicians were allocated to white coat-wearing consultations (*n* = 328), and casual attire-wearing consultations (*n* = 304). There was no difference in CARE Measure scores between white coat and casual primary care consultations overall (*p* = 0.162). Subgroup analysis of patient sex showed that CARE Measure scores of male patients were significantly higher in the Casual group than in the White coat group (adjusted *p*-value = 0.044). There was no difference in female patient scores between White coat and Casual groups (adjusted *p*-value = 1.000).

**Conclusions:**

This study demonstrated that physician attire (white coat or casual attire) in a primary care setting did not affect patient-perceived relational empathy overall. However, male patients of physicians wearing casual attire reported higher physician empathy. Although empathy cannot be reduced to simple variables such as attire, white coats may have a negative effect on patients, depending on the context. Family physicians should choose their attire carefully.

**Trial registration:**

Japanese University Hospital Medical Information Network (UMIN-ICDR). Clinical Trial identifier number UMIN000037687 (Registered August 14, 2019, https://upload.umin.ac.jp/cgi-open-bin/ctr_e/ctr_view.cgi?recptno=R000042749). The study was prospectively registered.

## Background

Most patients report that physician attire is important and associated with their satisfaction with care [1–3]. Patient preference for physician attire is influenced by age, locale, setting, and context of care [1, 3–8]. Reports from several countries suggest that patients prefer primary care physicians to wear white coats [4–6]. However, in some countries, most patients who visit a family physician (FP) no longer consider white coats a powerful symbol [8]. Previous research in Japan shows that most patients prefer physicians to wear white coats in a primary care setting [1]. However, one study found that some family medicine specialists certified by the Japan Primary Care Association (JPCA), which was established in 2010 following the merger of three primary care academic societies, preferred non-white coat attire, because they felt that casual attire allowed more empathetic interactions with patients [9]. However, there are no studies on whether FP attire influences relational empathy as perceived by patients in primary care settings.

Empathy contributes to effective general practice consultations [10] and has many beneficial effects in terms of health care, such as improved patient satisfaction, better medication adherence, greater patient enablement, and better clinical outcomes [11–15]. The identification of specific nonverbal behaviors that enhance patient-perceived relational empathy may be important for building efficient therapeutic relationships and optimizing patient health outcomes [15–18].

One study in a traditional medical clinic in Korea showed that patient-perceived empathy was substantially higher when physicians wore white coats and traditional dress than when they wore casual attire and suits [19]. However, a United States study of a large online sample in an analog medical context that manipulated physician nonverbal behaviors showed that patient-perceived empathy was affected by nonverbal communication (e.g., eye contact), not by physician white coat attire [17]. There is also evidence that, compared with male participants, female participants perceive doctors who express brusque nonverbal behavior as having low empathy. Empathy is a complex, multidimensional phenomenon that includes several functional processes, such as emotion recognition, emotional contagion, and emotion priming [20]. Empathy is also context-sensitive in patient–physician relationships [21]. Japan has a very unique culture that relies heavily on nonverbal and implicit communication [1, 22, 23]; thus, physician attire may play a more important role in patient–physician relationships in Japan than in other countries. In Japan, most patients prefer physicians to wear a white coat because it is considered professional or hygienic [24]. Conversely, white coat attire, with its connotations of professionalism, can be a symbol of a doctor’s paternalism, which may negatively influence the “human” aspects of medical care [25, 26]. It remains to be established whether FP dress style is associated with the perception of empathy in patient–physician relationships in Japan.

In this study, we investigated the use of alternating dress styles (casual attire vs. white coats) in FP practice to compare patient-perceived empathy, assessed using the Consultation and Relational Empathy (CARE) Measure. In addition, we tested previous findings [17] of a difference in perceived empathy between male and female patients.

## Methods

### Setting and study design

This trial was a multicenter, prospective, non-blind, controlled study conducted at primary clinics in Japan. We contacted 10 primary care clinics in the Tokai region of Japan and seven FPs in five clinics (four private clinics and one public clinic) agreed to participate in the study. The five clinics that declined to participate were all private clinics; the main reason given for declining was that staff expected to be very busy because of the high care demand over the winter period. The experience of the seven FPs who participated in the study ranged from 8 to 46 years. One FP was female. Five FPs were JPCA certified. The FPs’ usual attire was white coats (3), casuals (3), or scrubs (1).

Of the five clinics that participated, three had an appointment system. Patients made appointments by visiting the clinic in person, through the Internet, or by phone. Patients were not informed about the study at this stage. We recruited consecutive new patients (aged ≥ 20 years) in the clinics immediately after their consultation. New patients were defined as those who had not visited the clinic for 6 months or more. We excluded patients with conditions that may have been affected by a request for participation (e.g., anxiety disorder, serious infection, terminal illness) and those who could not complete the assessment independently because of their condition (e.g., dementia, blindness, deafness). We also excluded patients who visited the clinics for routine health checks or vaccinations, because the CARE Measure was originally developed in the context of the therapeutic relationship during one-on-one patient–clinician consultations [27].

**Fig. 1:**
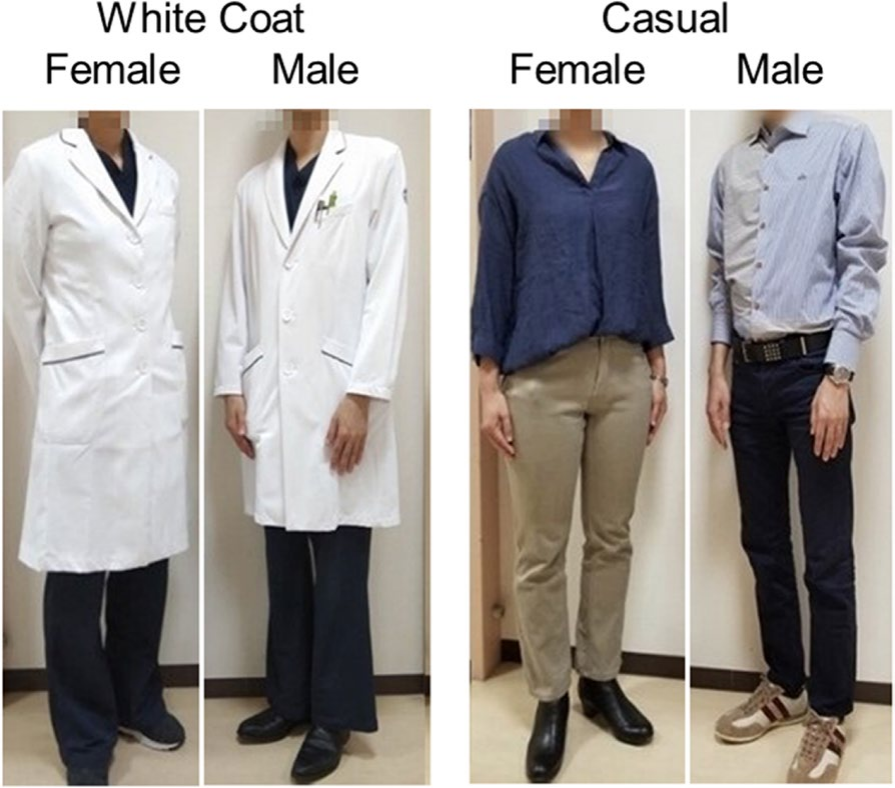
Photographs of model male and female physicians in white coats and casual attire

The study period was from October 2019 to April 2020. Each FP was asked to conduct their consultations wearing a white coat (White coat condition) or casual attire (Casual condition) on alternate weeks. FPs in the White coat group wore a white coat (Fig. 1); the wearing of undershirts, scrubs, and ties was not regulated. FPs in the Casual group wore a collared shirt without a white coat or a tie. We did not regulate the wearing of trousers, skirts, or shoes. The wearing of a facemask was fixed for each FP during the study period (two FPs wore facemasks; five FPs did not wear facemasks), because it has a negative effect on patient-perceived empathy [28]. At the end of each consultation, the FP invited the patient to fill out a questionnaire. If the patient agreed to participate, the reception staff gave them a questionnaire and explained it to them as required. Completed questionnaires were either mailed to the researchers or handed in to the reception staff in a sealed envelope. Survey participants were compensated for their time.

### Outcome

The primary outcome was the difference in scores on the Japanese CARE Measure between the White coat and Casual conditions. The CARE Measure is a widely used patient-reported measure of empathy that has demonstrated validity and reliability [29]. The CARE Measure was first developed in English [27] and has been translated into Japanese and validated in that language. The Japanese CARE Measure can effectively differentiate between doctors in terms of patient-rated empathy [30, 31]. Patients rated the 10 questions on the CARE Measure from 1 (“poor”) to 5 (“excellent”); there was also a “not applicable” option. In the case of missing or “not applicable” responses, the total CARE value was calculated by multiplying the average score for each item by 10. The total possible score range was 10 to 50. The questionnaire also recorded demographic and social information, including age, sex, marital status, education level, employment status, nature of the problem, and presence of chronic diseases.

### Sample size

A previous study found an average score on the Japanese CARE Measure for general practitioners of 38.41 (standard deviation 8.60) [30]. We estimated that 676 patients would be needed to detect a 2-point difference in CARE score, which is sufficient to detect a small to moderate standardized effect size using a two-tailed significance test with a power of 90% and an alpha level of 0.05. At least 38 consultations per doctor were required for the Japanese CARE Measure to differentiate between individual FPs on CARE score [31]. To detect significant CARE score differences in wearing a white coat or wearing casual attire, it was calculated that 100 consultations per FP (a total of 700) were needed.

### Statistical analysis

To test the effect on CARE Measure scores of wearing a white coat or casual attire during clinical consultations, we used a linear mixed model. This allowed us to adjust for cluster effects in patients nested within doctor, as well as potential confounding effects from patient demographic variables, such as age and sex of patients, and doctors’ sex and years of clinical experience. We also used a linear mixed model with Bonferroni adjustment to analyze the CARE score difference between the sexes, as previous research indicates a gender difference in empathy [17]. To examine the effect of sex on the primary outcome, we conducted a subgroup analysis of multiple comparisons by sex. Only nominal *p*-values less than 0.025 (= 0.05 divided by 2) were judged to be statistically significant. Statistical analyses were performed using SPSS software (version 26.0, IBM Corporation, Armonk, NY, USA).

## Results

### Study subjects

**Fig. 2:**
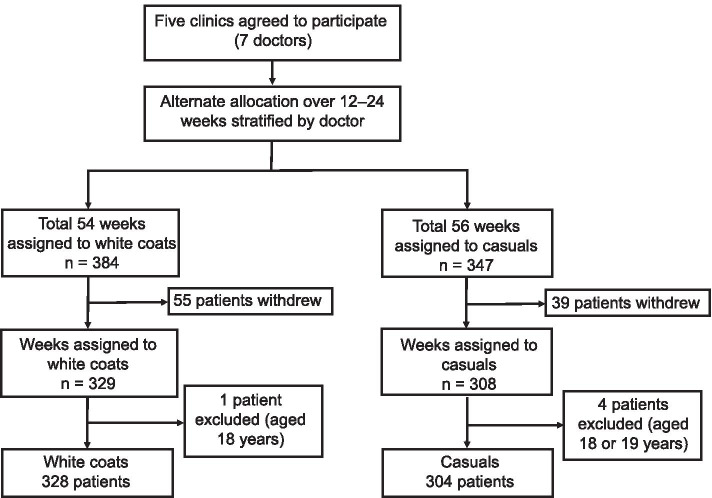
Flow diagram for trial participants

A total of 731 patients were consecutively recruited to the study; 637 patients (87.1%) submitted questionnaires (mailed: 94; handed in: 543). Data for five patients were excluded from the analysis because of the patients’ age, leaving 632 questionnaires (86.5%) (Fig. 2). Four doctors from two clinics terminated the study early because of the COVID-19 pandemic [32]. The number of participating patients per doctor ranged from 49 to 113 (median: 100, mean: 91) for the seven doctors. Mean CARE Measure scores for each doctor ranged from 31.8 to 41.6.

### Primary outcome

Patient characteristics are shown in Table 1. The two groups were evenly matched for most variables, although there was a higher percentage of females in the Casual group than in the White coat group (*p* = 0.012). Of 632 patients, the mean CARE score for the White coat group was 37.67 (95% confidence interval [CI]: 33.64–41.69) and that for the Casual group was 38.60 (95% CI: 34.60–42.60) (*p* = 0.162).

**Table 1:** Patient demographic characteristics

	White coat	Casual	*p*-value
	No. (%)	No. (%)	
Total	328	304	
Age (years)			
≤ 39	157 (47.9)	142 (46.7)	
40–69	152 (46.3)	133 (43.8)	
≥ 70	15 (4.6)	21 (6.9)	
Missing	4 (1.2)	8 (2.6)	
Sex			
Men	129 (39.3)	148 (48.7)	< 0.05
Women	196 (59.8)	148 (48.7)	< 0.05
Missing	3 (0.9)	8 (2.6)	
Marital status			
Single	97 (29.6)	79 (26.0)	
Married	205 (62.5)	203 (66.8)	
Separated	17 (5.2)	11 (3.6)	
Divorced	4 (1.2)	5 (1.6)	
Missing	5 (1.5)	6 (2.0)	
Education level			
Junior high school	20 (6.1)	13 (4.3)	
High school	107 (32.6)	86 (28.3)	
Vocational college	50 (15.2)	42 (13.8)	
Junior college	35 (10.7)	34 (11.2)	
University	100 (30.5)	108 (35.5)	
Graduate school	11 (3.4)	15 (4.9)	
Missing	5 (1.5)	6 (2.0)	
Employment status			
Employed (full- or part-time, including self-employed)	259 (79.0)	237 (78.0)	
Unemployed or looking for work	4 (1.2)	6 (2.0)	
Retired from paid work	11 (3.4)	13 (4.3)	
Unable to work owing to long-term sickness or disability	3 (0.9)	2 (0.7)	
Looks after the home/family	37 (11.3)	22 (7.2)	
At school or in full-time education	9 (2.7)	14 (4.6)	
Missing	5 (1.5)	10 (3.3)	
Nature of the problem			
New (acute) illness	269 (82.0)	244 (80.3)	
Old (chronic) illness	46 (14.0)	41 (13.5)	
Missing	13 (4.0)	19 (6.3)	
Presence of chronic diseases			
None	221 (67.4)	203 (66.8)	
All	107 (32.6)	101 (33.2)	

### Sex subgroup analysis

**Fig. 3:**
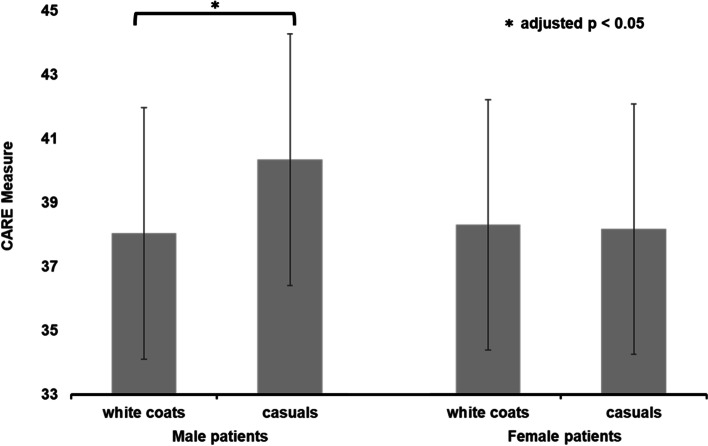
Graph showing the effect of attire and sex on CARE scores. Male patients in the Casual group (40.34) had a significantly higher CARE score than male patients in the White coat group (38.03) (adjusted *p*-value = 0.044). There was no difference in CARE score for female patients in the Casual (38.17) and White coat (38.30) groups (adjusted *p*-value = 1.000). CARE: Consultation and Relational Empathy Measure

In the linear mixed model analysis (adjusting for cluster effects of patients nested within doctor, age, sex of patients, and years of clinical experience and sex of doctors, and sex nested within attire), the p-value of the interaction between sex and attire was 0.072. The regression coefficient for sex was 0.027. We conducted a linear mixed model analysis for sex using the Bonferroni adjustment for 619 patients who provided full sociodemographic information, including sex and age. Figure 3 shows the sex difference between the White coat and Casual groups. The mean CARE score for males in the Casual group was significantly higher than that for males in the White coat group (40.34 vs. 38.03, adjusted *p*-value = 0.044). We found no difference between the mean CARE scores for females in the Casual group (38.17) and females in the White coat group (38.30) (adjusted *p*-value = 1.000). To explore the effect of age, we analyzed two groups of patients, one above and one below the average age, but there was no statistically significant difference between the two groups (adjusted *p*-value = 1.000).

## Discussion

This is the first multicenter, quasi-randomized controlled trial to examine the effect of physician attire (a white coat or casual attire) on patient-perceived relational empathy. We found no difference in empathy between the White coat and Casual attire conditions overall. However, the wearing of a white coat during FP consultations had a significant negative effect on male patients’ perceived empathy.

Previous studies in Japan [1, 24] have shown that most patients prefer Japanese FPs to wear white coats. It is likely that patients whose expectations are met in terms of their physician’s attire will experience more empathy in the therapeutic relationship. However, we found no difference in patient-perceived relational empathy between the White coat and Casual attire conditions. There are several possible patient-related reasons for this result. First, previous research indicates that FP clothing is a nonverbal cue that is perceived less frequently by patients compared with tone of voice, eye contact, and facial expressions [33]. Therefore, FPs’ choice of dress did not contribute substantially to empathy as perceived by patients. Second, a previous study [34] found that more tenseness was reported by new patients in a White coat group than in a Casual group, which suggests that the use of non-white coat attire in patient consultations may help to establish smoother patient–physician relationships. Third, modern patients have become more accustomed to physicians not wearing white coats, as increasing numbers of doctors do not wear white coats owing to concerns about contamination [9, 35–37]. From the physician’s perspective, a white coat confers professional identity at the expense of personhood, and so is not necessarily empathetic [35]. Our results differed from previous research in Korea which showed that patients’ perception of empathy was substantially higher when a traditional Korean medicine doctor wore a white coat or traditional attire than when they wore casual attire or suits [19]. Patient-perceived empathy may differ according to cultural differences and type of medical professional.

We also found that male patients were significantly more affected than female patients by perceived physician empathy when their physicians wore casual attire. There was a 2-point difference in the CARE Measure score, which is greater than the difference observed in previous studies with and without facemasks [28]. In a previous study that investigated gender differences in an emotion attribution task using functional magnetic resonance imaging, women and men relied on different strategies when assessing their own emotions in response to other people [38]. Previous research using the CARE Measure has also shown that female patients are more attuned than male patients to empathy signals such as lack of eye contact and unequal eye-levels [17]. Women are generally more sensitive than men to empathy and the feelings of others [21, 39]. Women are faster and more accurate at recognizing facial expressions than men [20], better at recognizing emotions, and express themselves more easily [39]. Female patients may be affected by features that are more salient than physician attire, such as tone of voice, eye contact, and facial expression [33]. However, the empathetic responses of male patients tend to be more influenced by contextual cues than those of female patients [20]. Men are also more responsive to threatening cues (dominant, violent, or aggressive cues) [39]. A white coat may be perceived as indicating medical paternalism [40], and so may affect the perceived empathy of male patients more than that of females. Although intriguing, further research is needed to explore such differences between male and female patients, as this was a secondary analysis in the present study.

This is the first multicenter, prospective controlled trial in primary care clinics to explore the differential effect of wearing a white coat or casual attire on empathy. One strength of the present study is that, to reduce information bias (and with the permission of our ethics committee), we explained to patients that the research was about empathy, but did not reveal that we were investigating the effect of physician attire. Our study has several limitations. First, for pragmatic reasons, patients were allocated on a weekly basis and there was no randomization. Second, the study design meant that the study was non-blind. Third, we did not reach the target sample size because we had to terminate the study early owing to the COVID-19 pandemic. This makes it difficult to draw firm conclusions from the findings. Fourth, a previous study identified a weak positive association between CARE score and consultation length, satisfaction with consultation length, and how well the patient knew the doctor [30]. We did not evaluate consultation length and satisfaction with the length, so we could not adjust the results. However, as our study targeted new patients, it was unlikely that the findings were affected by how well the patient knew the doctor. Fifth, the FPs in this study may not necessarily be representative of all Japanese FPs. Certification of FPs is changing in Japan. The JPCA began to certify FPs as “JPCA-certified family physicians” in 2010 [41] and the number of JPCA-certified FPs was only 900 as of September 30, 2020 [42]. From 2018, the certification changed to be a specialty based on the acquisition of general practitioner board certification [43, 44]. Therefore, most physicians currently working as FPs are not well-trained certified FPs and do not necessarily follow the global standard of primary care physicians [45]. For these reasons, statistical data for physicians working as FPs are not available. However, the participants of this study currently work as FPs, and we believe that they are fairly representative of FPs in Japan. Of the seven FP participants, six work in private clinics; this is close to the national situation, as more than 95% of medical clinics in Japan are private clinics [46]. Sixth, we did not regulate clothing worn under the white coat. This may have been a confounding variable, because a patient’s impression of a physician changes according to what the physician wears under his or her white coat [1]. Seventh, the effect of physician sex was not assessed because only one female physician participated in this study (below our target number for females). As perceived changes in facial expression are affected by the gender of both the source and recipient [47], more research is needed on this topic.

The present results suggest that physicians should be advised that wearing a white coat or casual attire does not have an overall effect on the establishment of patient–physician relationships in Japan, but that casual attire may have a positive effect on male patients. Additionally, white coat attire is associated with several problems, such as white coat hypertension [48, 49] and bacterial dissemination [36, 37]. Given these findings, it is perhaps time for physicians to consider alternatives to white coat attire.

## Conclusion

We found no difference overall in the effect of white coats and casual attire on patient-perceived relational empathy in primary care consultations, but male patients of physicians who wore casual attire reported higher physician empathy. Empathy is a complex, multidimensional phenomenon that is context-sensitive in patient–physician relationships and cannot be reduced to simple variables such as attire. White coats may have a negative effect on patients, depending on the context. FPs should choose their attire carefully.

## Data Availability

The datasets generated and analyzed during the current study are not publicly available due to confidentiality but are available from the corresponding author on reasonable request.

## Abbreviations

CARE: Consultation and Relational Empathy
FP: Family physician
JPCA: Japan Primary Care Association
